# Triptolide inhibits epithelial ovarian tumor growth by blocking the hedgehog/Gli pathway

**DOI:** 10.18632/aging.205110

**Published:** 2023-10-17

**Authors:** Lanyan Hu, Mai Gao, Huifu Jiang, Lingling Zhuang, Ying Jiang, Siqi Xie, Hong Zhang, Qian Wang, Qi Chen

**Affiliations:** 1 Department of Obstetrics and Gynecology, The Second Affiliated Hospital of Nanchang University, Nanchang 330006, Jiangxi, P.R. China; 2 Huankui Academy of Nanchang University, Nanchang 330036, Jiangxi, P.R. China

**Keywords:** ovarian cancer, hedgehog, triptolide, Gli1, Gli2

## Abstract

Epithelial ovarian cancer (EOC), the most predominant subtype of ovarian cancer (OC), involves poor prognosis and exhibits high aggression. Triptolide (TPL), like other Chinese herbs, has historically played a significant role in modern medicine. The screening system based on Gli-dependent luciferase reporter activity assessed the effects of over 800 natural medicinal materials on hedgehog (Hh) signaling pathway activity and discovered that TPL had an excellent inhibitory effect on Hh signaling pathway activity. However, the significance and mechanism of TPL involvement in regulating the Hh pathway have not been well explored. Thus, this work aimed to understand better how TPL affects the Hh pathway activity, which, in turn, influences the biological behavior of EOC. Our findings observed that Smo agonist SAG-induced EOC cell proliferation, migration, and invasion were drastically reversed by TPL in a concentration-dependent pattern. Further evidence suggested that TPL promotes the degradation of Gli1 and Gli2 to inhibit the activity of the Hh signaling pathway by relying on Gli1 and Gli2 ubiquitination. Our *in vivo* studies also confirmed that TPL could significantly inhibit the tumor growth of EOC. Taken together, our results revealed that one of the antitumor mechanisms of TPL was the targeted inhibition of the Hh/Gli pathway.

## INTRODUCTION

Ovarian cancer (OC), one of the deadliest gynecological cancers, is also the eighth leading cancer cause of death in women [1]. Histologically, epithelial ovarian cancer (EOC) is the most prevalent subtype of OC and makes up > 90% of all cases [2]. The major difficulty in the treatment of OC lies in the lack of reliable screening methods and the non-availability of clinical symptoms in its initial stage, resulting in the diagnosis of most patients with advanced malignancy (stages III or IV) [3, 4]. Despite rapid advances in tumor cytoreductive surgery, platinum-based chemotherapy, targeted agents, and immunotherapy, the therapeutic effects of OC therapy remain unsatisfactory. Most OC patients relapse because of chemotherapy resistance and metastasis within 1.5 years, and estimates indicate that approximately 70% of these patients lose their lives within 5 years [1, 5–9]. Therefore, a pressing need exists to tackle numerous challenging issues related to metastasis, chemotherapy resistance, and recurrence in the treatment of OC.

Chinese herbs have been an indispensable source of modern drugs. At present, more than 70% of antitumor drugs come from natural products [10]. Triptolide (TPL), a compound isolated from Tripterygium wilfordii Hook f, has been utilized for centuries in traditional Chinese medicine [11]. TPL has attracted considerable attention for its wide-ranging effectiveness, such as its broad spectrum antitumor, antirheumatic, anti-fertility, immunosuppressive, antiangiogenesis, and anti-osteoporosis effects [12]. As early as 1972, the anti-cancer properties of TPL were found to induce apoptosis in leukemia, subsequently, TPL was proven to exhibit drastically antitumor properties in breast cancer [13], gastric cancer [14], gallbladder carcinoma [15], osteosarcoma [16], neuroblastoma [17], OC [18], prostate cancer [19], and melanoma [20]. Nevertheless, the molecular mechanism by which TPL exerts its anticancer activities in OC is not well understood.

The hedgehog (Hh) pathway is essential for numerous key developmental events in the embryo, while its abnormal activation was closely correlated with many malignancies, including OC [21–23]. Abnormal activation of Hh signaling promotes carcinogenesis and enhances tumor malignant phenotypes of cancers, including proliferation, aggression, and chemotherapy and radiotherapy resistance [21, 24–26]. Three Hh homologs members have been recognized in mammals: Shh, Dhh, and Ihh, Shh has been deemed to be the most potent biological effects ligand of the Hh homologs [27]. When an Shh ligand binds to the Ptch1 receptor, Ptch1-mediated smoothing (Smo) inhibition is released, consequently, starting the canonical activation of the Hh pathway. Further, this activation of the Hh pathway promoted the nuclear accumulation of transcription factors Gli family (namely, Gli1, Gli2, and Gli3), triggering the expression of target genes like as Gli1, Ptch1, Bcl2, and FoxA2 [28]. Substantial effort has been directed toward treating this pathway-driven cancer because of the vital function of the Hh pathway in tumorigenesis and development. Most reported Hh inhibitors are regarded as the Smo and Gli antagonists. Targeted inhibition of Smo is a flexible process, whereas the mechanism acting on Gli members is complex, thus, Gli antagonists are not as widespread as Smo counterparts in clinical trials [29, 30]. However, Gli1 and Gli2 are the essential and ultimate effectors of the Hh pathway, and structural activation of either of them is crucial to the development of cancer. GDC-0449 and Sonidegib have received approval from the Food and Drug Administration to treat cancer, but like other targeted cancer medicines, these two Smo inhibitors have caused tumor relapse, primarily attributable to acquired resistance to Smo and the activation of the Hh non-classical pathway [31, 32]. With this research, we sought to explore whether one of TPL’s antitumor mechanisms is targeted suppression of the Hh pathway.

## RESULTS

### Triptolide inhibits EOC cells proliferation, migration, and invasion

**Figure 1:**
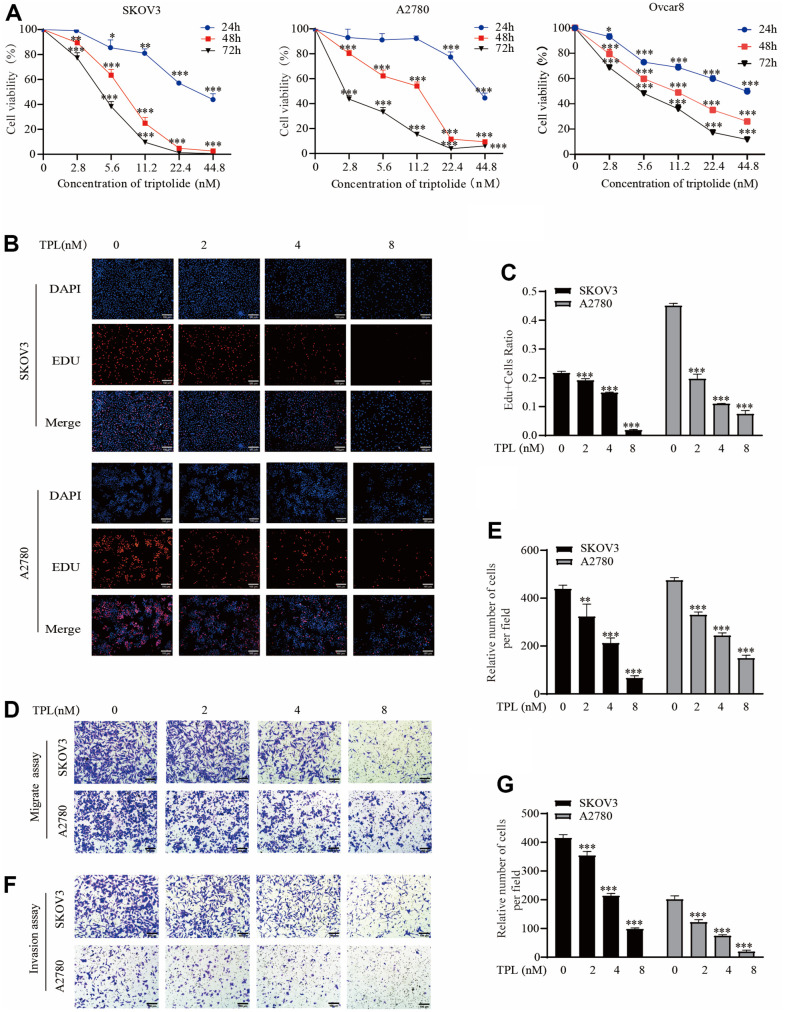
**Triptolide inhibits EOC cells proliferation, migration, and invasion.** (**A**) SKOV3, A2780, and Ovcar8 were incubated with TPL for 24, 48, or 72 h at various doses (0 – 44.8 nM). The cell cytotoxicity assays were detected using CCK-8 analysis and IC50 was calculated by GraphPad Prism software 8.0. (**B**, **C**) SKOV3 and A2780 cells proliferation after treatment with various doses of TPL by EdU incorporation assay, (**C**) quantification of EdU-positive cells were analyzed. (**D**, **E**) Migration assay showed TPL dampened migration ability of EOC cells, Graph (**E**) represents the positive cell’s number of migrations. Scale bar: 100 μm. (**F**, **G**) Invasion assays showed TPL dampened invasion ability of EOC cells, Graph (**G**) represents the positive cell’s number of invasions. Scale bar: 100 μm. All results were derived from three independent repeated experiments and represented by mean ± SD, * *p* < 0.05, ** *p* < 0.01, and *** *p* < 0.001 vs. control.

Initially, we performed the CCK8 experiment to examine the impact of TPL on the cytotoxicity of the EOC cells SKOV3, A2780, and Ovcar8. The experiment on cell cytotoxicity showed that the IC_50_ of TPL on SKOV3 cells was 38.26 ± 5.83, 7.06 ± 1.13, and 3.4 ± 1.11 nM at 24, 48, and 72 h, respectively. For A2780 cells, the IC_50_ of TPL was 37.59 ± 5.61, 7.83 ± 2.26, and 3.04 ± 1.29 nM at 24, 48, and 72 h. The IC_50_ of TPL on Ovcar8 cells at 24, 48, and 72 h were 36.92±3.96, 10.93±0.08, and 5.62±0.34 nM (Figure 1A). The sensitivities to TPL between the three EOC cells varied slightly. This result indicated that TPL was cytotoxic against EOC cells in a time-and dose-dependent manner. Then, we chose concentrations 2, 4, and 8 nM of TPL for a follow-up study in EOC cells. The antiproliferative activity of TPL was further evaluated with an EdU incorporation assay. The EDU assay findings also showed that the anti-proliferation ability of TPL was markedly enhanced with increased doses (Figure 1B, 1C).

As shown in supporting information Figure 1D–1G, the effects of TPL on the aggression of EOC cells were ascertained by using Transwell migration and Invasion assay separately. These observations confirmed that TPL greatly dampened the aggression of EOC cells in a dose-dependent pattern.

### Triptolide inhibits the Hh pathway activity in EOC cells

**Figure 2:**
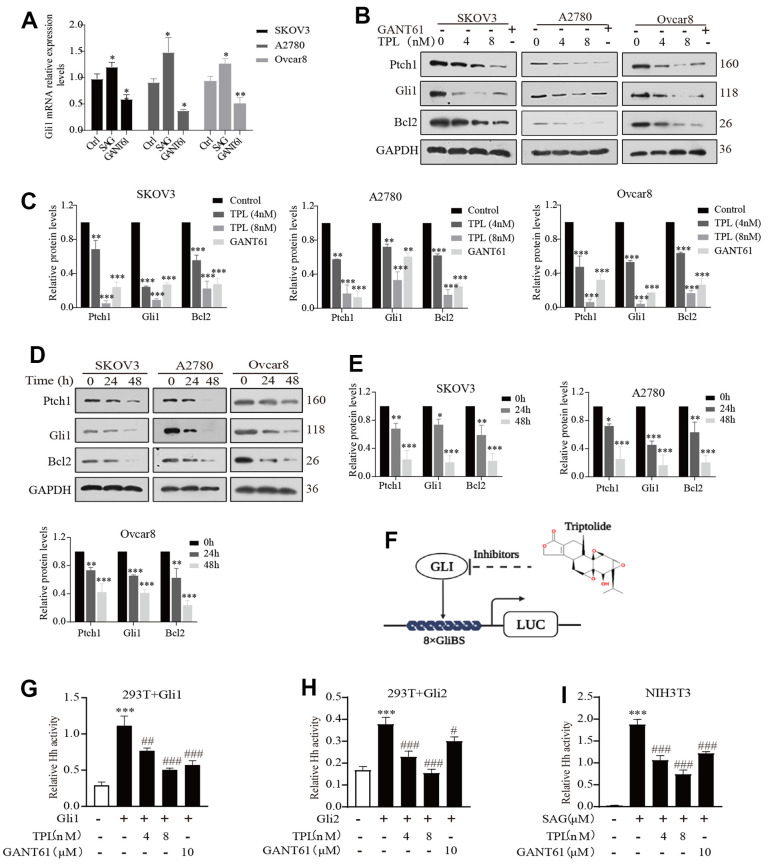
**Triptolide inhibits the Hh pathway activity in EOC cells.** (**A**) SAG (1 μM) and GANT61 (10 μM) effects on Gli1mRNA expression EOC cells *in vitro*. (**B**, **C**) Western blot was used to measure the protein levels of Ptch1, Gli1, and Bcl2 in SKOV3, A2780, and Ovcar8 cells for 48 h after TPL or GANT61 administration, GANT61 was used as a positive control, GAPDH was used for normalization. (**D**, **E**) The time-dependent effect of TPL (4 nM) on the protein levels of Ptch1, Gli1, and Bcl2 was carried out by Western blot, and GAPDH was used for normalization. (**F**) The schematic composite screen is used to recognize small molecular Gli antagonists, the molecular structure of TPL is given in the upper right corner. (**G**, **H**) The HEK293T cells were transiently transfected with 8xGliBS-Luc, pTK-Renilla, and Gli (Gli1 or Gli2) plasmids for 24 h, then treated with TPL or GANT61 for 36 h, and then the fluorescence value is determined according to the instructions. (**I**) The NIH3T3 cells were transiently transfected with 8xGliBS-Luc and pTK-Renilla plasmids for 24 h, then treated with various compounds for 36 h. All results were derived from three independent repeated experiments and represented by mean ± SD, * *p* < 0.05, ** *p* < 0.01 and *** *p* < 0.001 vs. control; # *p* < 0.05, ### *p* < 0.01, ### *p* < 0.001 vs. Gli1, Gli2, or SAG group.

The Hh pathway-related proteins, including Ptch1 and Gli1 proteins, have been expressed in primary ovarian tumors and cell lines, furthermore, the abnormal activation of this pathway contributes to the malignancy of OC [33, 34]. Significantly, however, Tom et al. found that the activity of the Hh pathway was inhibited in medulloblastoma cells cultured *in vitro* [35, 36]. Thus, we first detected the activity of the Hh signaling pathway in EOC cells cultured *in vitro* and found that there was activation of the Hh signaling pathway in EOC cells *in vitro* (Figure 2A). Subsequently, we confirmed that TPL could down-regulate the expression of Ptch1, Gli1, and Bcl2 proteins in a time-and-dose-dependent pattern by Western blot (Figure 2B–2E). Among the proteins, Gli1, Ptch1, and Bcl2 could be used as signals of abnormal activation in the Hh pathway [37].

To further examine whether TPL could restrain Hh signaling activity, we focused on the Gli proteins because they are the primary and final effectors of the Hh signaling pathway. However, considering Gli1 and Gli2 share high homology, it was presumed that effective Gli1 inhibitors would also target Gli2 [38]. Luciferase reporter assay showed that TPL and GANT61 suppressed the Gli1- and Gli2- mediated transcription after transient transfection of Gli1 or Gli2 in HEK293T cells (Figure 2F, 2G). Additionally, we used a mouse NIH3T3 cell line with endogenous Hh signaling ability and confirmed that TPL and GANT61 drastically restrained the Gli-responsive reporter activity provoked by the 1μM Smo agonist SAG (Figure 2H). Taken together, these data illustrated that TPL could markedly suppress the Hh pathway activity.

### Triptolide inhibits SAG-induced EOC cells proliferation, migration, and invasion

**Figure 3:**
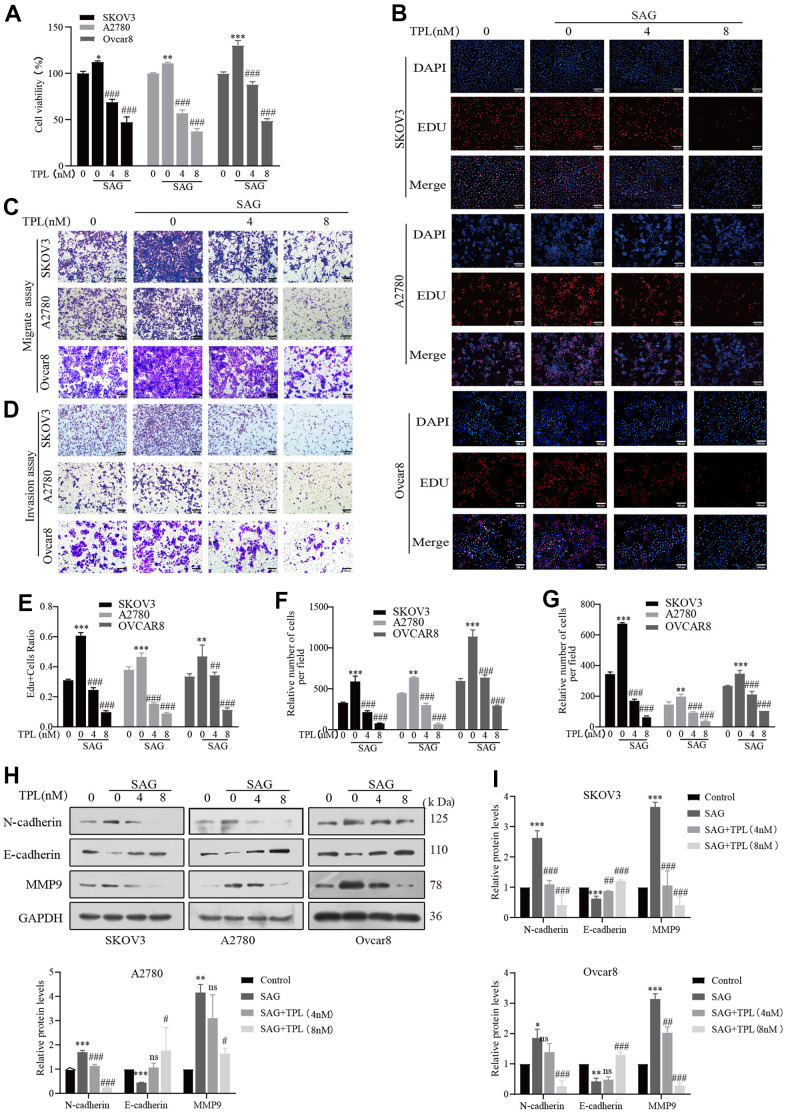
**Triptolide inhibits SAG-induced EOC cells proliferation, migration, and invasion.** (**A**, **B**) TPL inhibited the cell proliferation provoked by SAG (1 μM). (**C**) Migration assay showed that TPL restrains the SAG-induced migration ability of SKOV3, A2780, and Ovcar8 cells. (**D**) Invasion assay showed that TPL inhibits SAG-induced invasion ability. (**E**) The quantification of EdU-positive cells was analyzed. Graph (**F**) represents the positive cell's number of migrations. Graphs (**G**) represent the positive cell's number of invasions. (**H**, **I**) The effects of SAG on the protein levels of MMP9, E-cadherin, and N-cadherin protein, and TPL could reverse the results produced by SAG, and GAPDH was used for normalization. Scale bar: 100 μm. All results were derived from three independent repeated experiments and represented by mean ± SD, * *p* < 0.05, ** *p* < 0.01 and *** *p* < 0.001 vs. control; # *p* < 0.05, ## *p* < 0.01 and ### *p* < 0.001 vs. SAG group; ns, no significance.

To establish whether TPL could restrict tumor growth by preventing the Hh pathway, we applied SAG to further stimulate this pathway in EOC cells. Both CCK8 and EDU experiments revealed that SAG effectively promoted the proliferation of EOC cells, but TPL treatment dramatically dampened SAG-mediated cell proliferation (Figure 3A, 3B, 3E). Similar phenomena were seen in the Transwell migration and Invasion experiment. SAG remarkably enhanced the capacity of EOC cells A2780, SKOV3, and Ovcar8 to migrate and invade, but TPL effectively suppressed SAG-induced cell migration (Figure 3C, 3F), and invasion ability (Figure 3D, 3G). To further investigate the related markers of invasion, we utilized Western blot analysis to evaluate the protein levels of the epithelial-mesenchymal transition (EMT) markers N-cadherin, E-cadherin, and MMP9, our results showed that SAG upregulated the expression of N-cadherin and MMP9 and downregulated the expression of E-cadherin, but TPL notably reversed these results in a concentration-dependent way (Figure 3H, 3I). These findings implied that the TPL-mediated anti-EOC mechanism should involve the Hh signaling pathway.

### Triptolide overcomes SMO mutant-induced drug resistance

**Figure 4:**
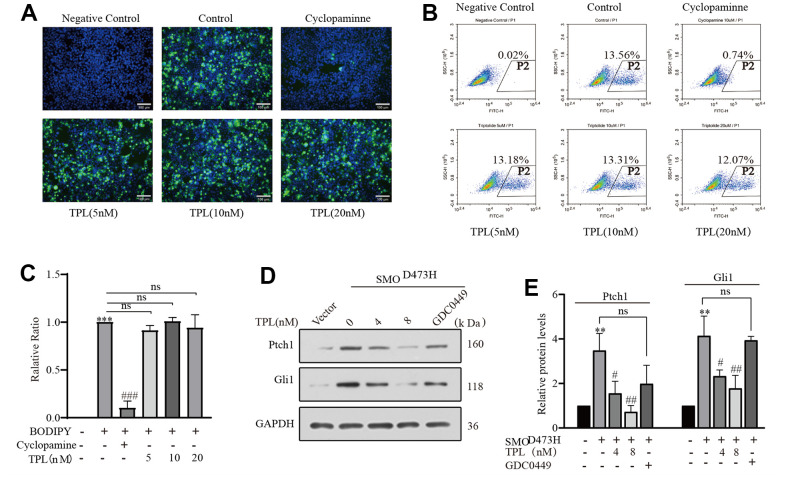
**Triptolide overcomes SMO mutant-induced drug resistance.** (**A**–**C**) The BODIPY-cyclopamine (5 nM) competitive binding assay was separately examined by fluorescent microscope and flow cytometry, Cyclopamine (5 μM) was used as a positive control. (**C**) Quantification of flow cytometry following BODIPY-cyclopamine competitive binding assay analysis was exhibited. Scale bar: 100 μm. (**D**, **E**) Western blot was used to detect the effect of TPL on the expression of Gli1 and ptch1 in NIH3T3 cells after overexpression of SMO473H, and GDC0449 (1 μM) was used to control. All results were derived from three independent repeated experiments and represented by mean ± SD, * *p* < 0.05, ** *p* < 0.01 and *** *p* < 0.001 vs. vector; # *p* < 0.05, ## *p* < 0.01 and ### *p* < 0.001 vs. BODIPY-cyclopamine or SMO473H group; ns, no significance.

The above results proved that TPL markedly restrained SAG-induced Hh pathway activity. Come to talk theoretically, every core member of the Hh signaling pathway could be used as a target for cancer therapy. At present, the majority of clinically available inhibitors of the Hh pathway directly targeted Smo or its downstream proteins Gli. To investigate whether Smo is the target gene of TPL, we conducted the BODIPY-cyclopamine competition assay and detected HEK293T cells overexpressing Smo by fluorescence microscopy and flow cytometry [39], using the Smo inhibitor Cyclopamine as a positive control. Notably, TPL did not compete with BODIPY-cyclopamine for the Smo receptor, whereas cyclopamine effectively competed with BODIPY-cyclopamine for the Smo receptor (Figure 4A–4C). In addition, a mutation of Smo (SMO^D473H^) exhibited resistance to GDC-0449 therapy [38]. To further investigate whether the drug resistance caused by SMO^D473H^ mutation could be overcome, we successfully constructed the SMO^D473H^ mutant plasmid and verified it with Smo inhibitor GDC0449 (Supplementary Figure 1). On the contrary, when we transfected mutant Smo^D473H^ into NIH3T3 cells, the protein levels of Ptch1 and Gli1 were notably upregulated, these outcomes serve as reliable indicators of the activation of the Hh pathway, and such activation could be inhibited by TPL in a concentration-dependent pattern (Figure 4D, 4E). Accordingly, we posit that the depressant effect of TPL on the Hh signaling pathway probably not be Smo, of course, it may also be different from the binding site of cyclopamine and Smo. In such circumstances, thus, it is led us to highly suspect that TPL acts directly on Gli or downstream molecules of the signaling pathway in Smo.

### Triptolide blocks the Hh signaling pathway by interfering with Gli

**Figure 5:**
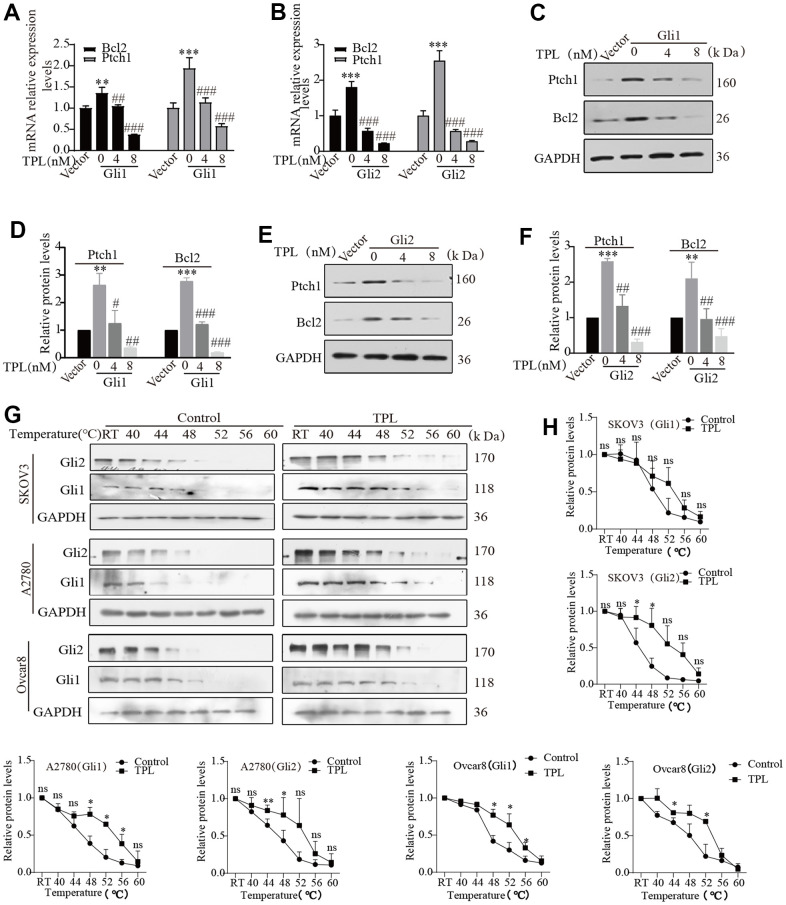
**Triptolide blocks the Hh signaling pathway by interfering with Gli.** (**A**–**F**) The HEK293T cells were transiently transfected with Gli1 or Gli2 plasmids for 24h, then incubated with different doses of TPL for 48 h, and then were conducted to qPCR and Western blot for Ptch1 and Bcl2 mRNA and protein levels. GAPDH was used for normalization. (**G**, **H**) CETSA was performed in SKOV3, A2780, and Ovcar8 cells, cells incubated with or without TPL (50 nM) for 4 h and then treated at different temperatures (RT, 40, 44, 48, 52, 56, 60° C), and protein levels of Gli1 and Gli2 were conducted by Western blot, GAPDH was used for normalization. All results were derived from three independent repeated experiments and represented by mean ± SD, * *p* < 0.05, ** *p* < 0.01 and *** *p* < 0.001 vs. vector or control; # *p* < 0.05, ## *p* < 0.01 and ### *p* < 0.001 vs. Gli1 or Gli2 group; ns, no significance.

The activation of the Hh pathway comprises both classical and non-classical pathways. For both pathways, all biological functions depend on the transcriptional effector Gli at their distal ends. Therefore, targeted inhibition of Gli transcription factors may represent a better choice for restraining the Hh pathway activity. To further investigate whether TPL inhibits the activity of the Hh pathway through targeted inhibition of Gli, we focused on investigating transcription factors Gli1 and Gli2, which primarily function as transcriptional activators to activate the Hh signaling pathways and exhibit high homology. Thus, Gli1 or Gli2 were transfected into the HEK293T cells and treated with or without TPL. The transfection efficiency was verified by Western blot (Supplementary Figure 2). RT-qPCR and Western blot experiments revealed that TPL therapy dramatically reduced the mRNA (Figure 5A, 5B) and protein expression (Figure 5C–5F) of target genes Ptch1 and Bcl2 in a concentration-dependent pattern. To provide further evidence for this observation, we next investigated whether TPL and Gli1 or Gli2 directly interacted to control the activity of the Hh pathway. The interaction between TPL and Gli1 or Gli2 protein was verified with a CETSA assay. Our results established that the EOC cells after TPL treatment had significantly higher thermal stability. Results from the CETSA assay suggested that TPL could directly bind Gli1 and Gli2 proteins (Figure 5G, 5H).

### Triptolide enhanced the protein degradation of Gli1 and Gli2 through ubiquitin-proteasome-dependent pathway

**Figure 6:**
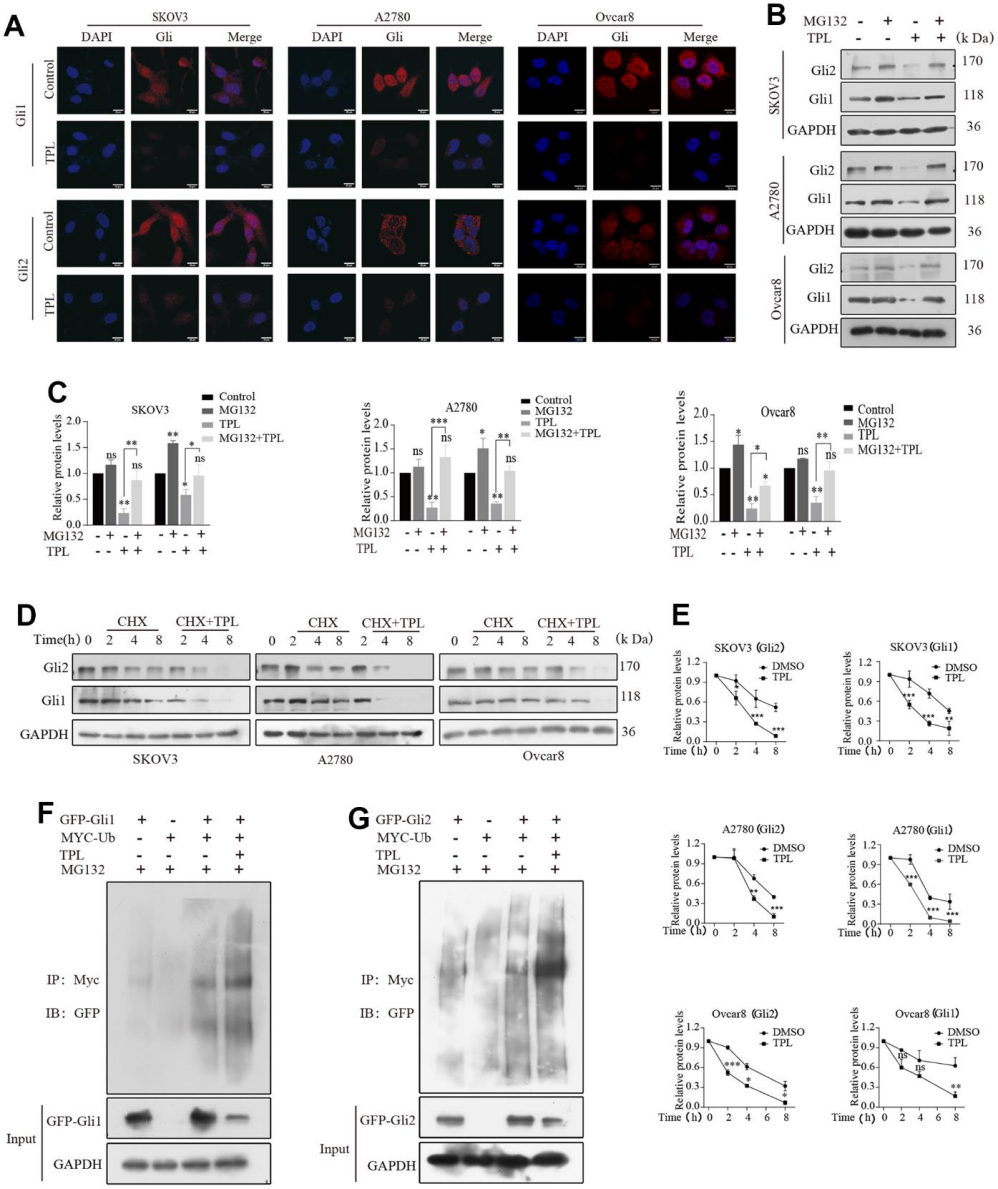
**Triptolide enhanced the protein degradation of Gli1 and Gli2 through the ubiquitin-proteasome-dependent pathway.** (**A**) TPL (4 nM) was applied to SKOV3 and A2780 cells for 24 h, and the results were separately detected using immunofluorescent assays with anti-Gli1 and anti-Gli2 antibodies. Scale bar: 20 μm. (**B**, **C**) SKOV3 and A2780 cells were incubated with TPL (4 nM) for 48h and then MG132 (10 μM) for 4h before harvesting and were subjected to Western blot for Gli1 and Gli2. (**D**, **E**) Cells were treated with cycloheximide (20 μM) with or without TPL (4 nM) and detected the protein levels of Gli1 and Gli2 by Western blot assay. (**F**, **G**) HEK293T cells were cotransfected with the Myc -Ub, Gli (GFP-Gli1 or GFP-Gli2), or the GFP control vector plasmids, and then the cells were further cultivated for 36 h with TPL after being cultured for 24 h. During the last 4h of TPL treatment, MG132 (10 μM) was added to every group. The protein lysates were examined by immunoprecipitation with an anti-MYC antibody, and the ubiquitinated protein levels were detected by Western blot in an anti-GFP antibody. All results were derived from three independent repeated experiments and represented by mean ± SD, * *p* < 0.05, ** *p* < 0.01, and *** *p* < 0.001 vs. control; ns, no significance.

As stated above, we established a close relationship between TPL with Gli1 and Gli2. Hence, we then sought to define the mechanistic basis of the action of TPL on Gli1 and Gli2 in greater depth. Since nuclear-cytoplasmic shuttling was considered to be the primary way to regulate the biological activity of Gli1 and Gli2, we first hypothesized TPL influences Hh signaling activity by acting on the nuclear localization of Gli1 and Gli2. The immunofluorescent assay revealed that Gli1 and Gli2 were considerably inhibited in both the nucleus and cytoplasm after TPL treatment, whereas the nuclear accumulation of Gli1 and Gli2 was unaffected (Figure 6A).

Considering that one of the main mechanisms for controlling Gli activity is proteasomal degradation [40]. we subsequently investigated whether TPL could also affect Gli activity through the proteasome degradation pathway. As displayed in Figures 6B, 6C, the TPL-induced decrease of the Gli1 and Gli2 proteins was recovered by treatment with proteasome inhibitor MG132. Furthermore, we studied the effect of TPL on the stability of Gli1 and Gli2 proteins treated with the protein synthesis inhibitor cycloheximide (CHX). The outcomes proved that TPL treatment dramatically accelerated the degradation of Gli1 and Gli2 proteins in the SKOV3, A2780, and Ovcar8 cells (Figure 6D, 6E). The ubiquitin-proteasome system (UPS), a crucial pathway of protein degradation, participates in the degradation of most proteins. Consequently, we were curious about TPL to assess its effect on the ubiquitination of Gli1 and Gli2. The results showed that TPL treatment dramatically downregulated the Gli1 and Gli2 protein levels and upregulated the ubiquitination levels of Gli1 and Gli2 (Figure 6F, 6G). Altogether, our study demonstrated that the UPS was responsible for the TPL-induced Gli1 and Gli2 protein degradation.

### Triptolide repressed tumor growth in A2780 xenografts

**Figure 7:**
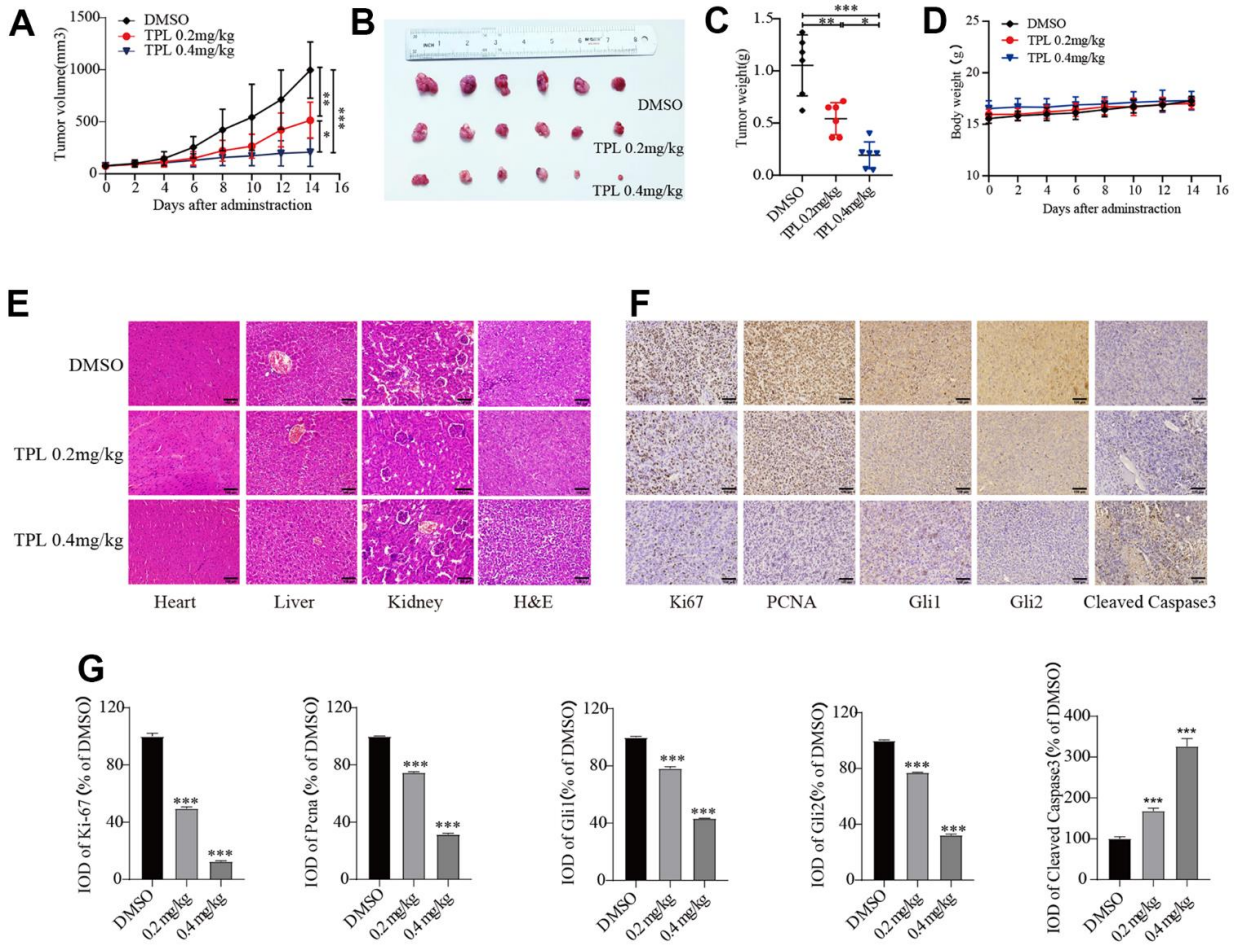
**Triptolide repressed tumor growth in A2780 xenografts.** (**A**) The tumor volume curve. (**B**) Tumor samples were shown. (**C**) Tumor weight. (**D**) Body weight of mice. (**E**) H&E staining was used to observe the toxic and side effects of the TPL. (**F**, **G**) The expressions of Ki67, PCNA, Gli1, Gli2, and Cleaved Caspase 3 in tumor tissues of each group were detected by IHC. Quantitative data are presented in graph (**G**). Scale bar: 100 μm. * *p* < 0.05, ** *p* < 0.01 and *** *p* < 0.001 vs. control.

Lastly, we further validated the impact of TPL on EOC *in vivo*. Three groups of mice (n=6) were given intraperitoneal injections of DMSO, TPL (0.2 mg/kg/d), or TPL (0.4 mg/kg/d). The tumor volume and final weight of treatment with TPL administration groups were remarkably lower in comparison to the DMSO group (Figure 7A–7C). The body weight of the TPL groups was not decreased significantly compared with the DMSO groups (Figure 7D). Major organs like the heart, liver, and kidney did not experience structural abnormalities as a result of TPL treatment, according to toxicity studies using H&E staining (Figure 7E). According to the above findings, TPL has a strong inhibitory effect on tumor growth and exhibits low toxicity in tumor-bearing mice at dosages of 0.2 and 0.4 mg/kg. IHC staining studies revealed that TPL treatment significantly boosted the expression of the apoptotic protein Cleaved Caspase 3, indicating that TPL treatment induced tumor cell apoptosis. In contrast, TPL treatment markedly reduced the expression of the proliferation-associated proteins PCNA and Ki-67 compared with the DMSO group, as expected (Figure 7F, 7G). In addition, we performed IHC staining of the Gli1 and Gli2 to understand whether TPL could restrict Hh pathway activity *in vivo*. Our research indicated that TPL therapy dramatically reduced Gli1 and Gli2 protein levels (Figure 7F, 7G). As a whole, the above outcomes confirmed that TPL could hinder the proliferation of EOC cells and the activity of the Hh pathway *in vivo*.

## DISCUSSION

TPL has received a great deal of attention for its ability to inhibit numerous cancers, but the antitumor mechanism of TPL is still far from clear. Multiple studies have indicated that the anticancer activity of TPL involves many potential mechanisms. Gao et al. showed that TPL could effectively depress the growth of colorectal cancer by reducing the expression of the RNA polymerase III target gene [41]. Besides that, Tan et al. have observed that TPL suppresses the malignant progression of platinum-resistant OC by acting on the PI3K/AKT/ NF-kB pathway [42]. In this research, we confirmed that TPL could greatly diminish the invasion and migration ability of EOC cells in a dose-dependent way. Therefore, these observations collectively indicate that TPL has an anti-EOC effect. Our previous research confirmed that the Hh signaling pathway boosts aggressive phenotypes of OC by regulating CD24 and MMP7 through Gli1 and Gli2, respectively [43–45]. These results contribute to a better understanding of the thought that inhibiting the Hh pathway should be an effective therapeutic measure for OC. Moreover, increasing evidence suggests that natural compounds play a crucial role in the prevention and treatment of cancer by regulating the Hh pathway [46]. In this study, we successfully confirmed that TPL could markedly suppress the activity of the Hh pathway.

EMT is a dynamic process in which epithelial cells lose their characteristics and acquire mesenchymal characteristics, which is mainly characterized by the decrease of epithelial markers (such as E-cadherin) and the increase of the expression of mesenchymal markers (such as N-cadherin), the decline of E-cadherin protein expression has been thought to be the most prominent feature of EMT [47]. Additionally, several studies have also found that the decrease of basement membrane may be an important reason for the progression of tumor metastasis, and matrix metalloproteinases (MMPs) could effectively degrade cell basement membrane [48]. Among them, MMP9 could destroy type IV collagen proteins in the extracellular matrix, which is the most abundant component of the basement membrane, so it also plays an important role in the invasion and metastasis of malignant tumors. Much evidence demonstrated that the overactivation of the Hh pathway was regarded to be closely connected to tumor growth and metastasis [49, 50]. Fortunately, our research results showed that TPL remarkably dampened the SAG-induced proliferation and aggression of EOC cells, and affected the expression of EMT-associated proteins and MMP9.

These above results strongly support the contention that TPL could exert its anti-EOC effect partly by inhibiting the activity of the Hh pathway. Preclinical studies on this pathway mainly focused on the inhibition of Smo and Gli. Regrettably, Most of the Smo inhibitors that have been used clinically have developed drug resistance due to Smo mutations, the non-canonical activation of the Hh signaling pathway, and the loss of primary cilia [29]. To present, several studies reported that around 50% of basal cell carcinomas resistant to Smo inhibitors have Smo mutations, Importantly, further analysis revealed that about 17% of basal cell carcinoma drug resistance found Smo^D473^ site mutation [51, 52]. In our work, we confirmed that TPL could effectively overcome the drug resistance caused by SMO^D473H^ mutation.

CETSA has become one reliable approach for evaluating target involvement in the physiological environment, and the binding of small molecules to protein targets is a crucial process to further understanding the mechanism of drug action [53]. Herein, the results from the CETSA assay suggested that TPL could interact with the Gli1 and Gli2. Our findings also support the research results of other works which established that the roles of Gli1 and Gli2 in diseases overlap to some extent because of their high homology and similarity in promoter recognition sequences, that is to say, developed inhibitors claim that compounds specific to Gli1 may be non-specific [30, 54] Note that the dynamic bidirectional transport of transcription factors between cytoplasm and nucleus is vital for their functions [55]. According to the majority of existing research, Gli proteins must be transported by cilia to be activated and transported to the nucleus. Nevertheless, investigations have revealed that the Gli2 protein could produce skin cancers even in the absence of primary cilia, thereby providing evidence that the Gli protein did not need cilia to reach the nucleus [55, 56]. Surprisingly, TPL treatment in this work did not affect Gli1 and Gli2 subcellular distribution. Proteasome inhibitor MG132 drastically recovered the Gli1 and Gli2 expression decreased by TPL, showing that the proteasome-dependent protein degradation mechanism was responsible for the TPL-induced Gli1 and Gli2 protein degradation. Furthermore, the rate of Gli1 and Gli2 degradation was increased under TPL treatment in EOC cells. Hence, TPL regulates the expression of Gli1 and Gli2 proteins by reducing the stability of those proteins at the posttranslational level. The UPS regulates the activity of transcription factor Gli in at least two ways. On one hand, the UPS destroys Gli to inhibit the Hh signaling response when the quantity of the Hh ligand is high. On the other hand, the UPS partially degrades Gli in the absence of Hh ligand stimulation, changing it from a transcriptional activator to an inhibitor, and consequently balancing the inhibition and activation of Gli [21, 57, 58].

**Figure 8:**
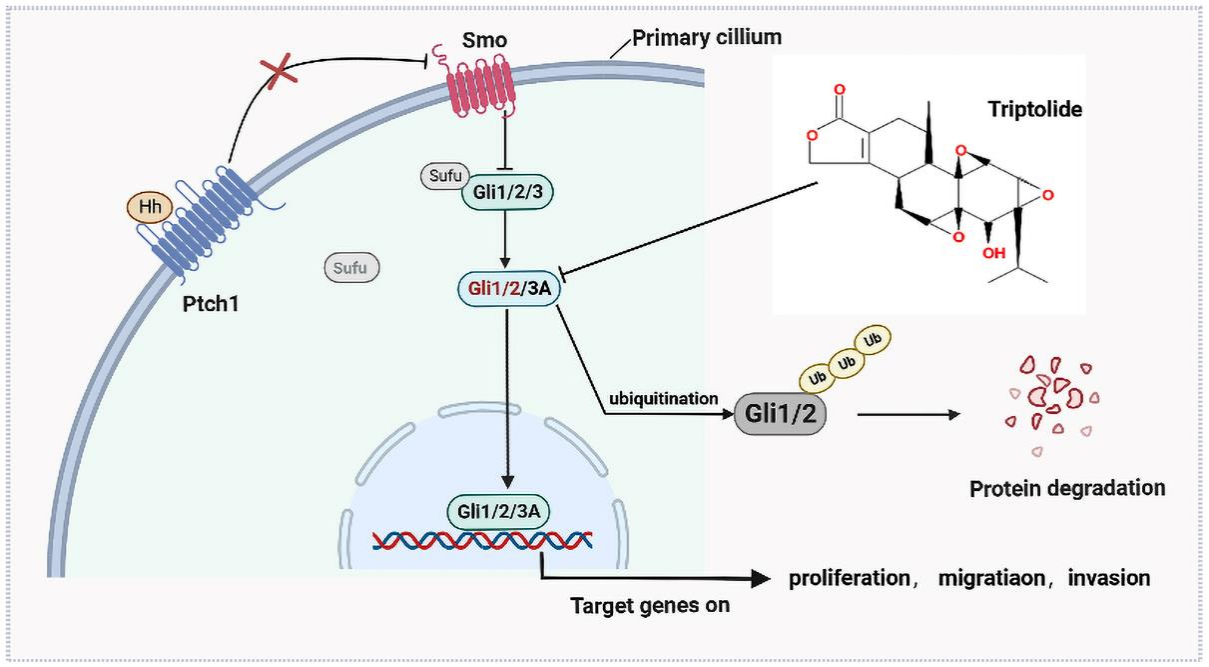
The antitumor mechanism of triptolide.

In conclusion, the aggregate data in this study demonstrated that SAG could further activate the Hh pathway to promote the proliferation and invasion ability of EOC cells, and this behavior could be abrogated by TPL. Additionally, we have also offered a newly discovered that TPL, as an inhibitor of the Hh pathway, plays a direct role by targeting Gli1 and Gli2 (Figure 8). Taking this new information into consideration, we concluded that our findings provided a novel molecular basis for TPL as a promising treatment for inhibiting EOC growth.

## CONCLUSIONS

This research elegantly indicated a novel molecular mechanism of TPL in the treatment of EOC. The occurrence and development of EOC are tightly correlated with the activation of the Hh pathway, our experiments *in vivo* and *in vitro* illustrated that TPL could suppress the proliferation and invasion of EOC by targeting the Hh pathway signaling and effectively prevent the progression of EOC.

## MATERIALS AND METHODS

### Material and antibodies

TPL was a purity of 98% purchased from J&K Scientific (USA) (CAS: 38748-32-2), dissolved in dimethyl sulfoxide (DMSO), and stored at -80° C. Gli antagonist GANT61 was purchased from Sigma-Aldrich (USA) (CAS: 500579-04-4), Smoothened antagonist GDC0449 (CAS: 879085-55-9) and Cyclopamine (CAS: 4449-51-8) and Smoothened agonist SAG (CAS: 912545-86-9) were obtained from selleckChem (USA). Antibodies against GAPDH, Gli2, GFP (50430-2-AP), MYC (16286-1-AP), and goat anti-rabbit/mouse IgG were purchased from ProteinTech Group (China), anti-Ptch1 (Zenbio, China), anti-Gli1 (Bioss, China), anti-Bcl2 (Abclonal, a19693, USA), anti-Cleaved Caspase 3 (Cell Signaling Technology #9664, USA), and the other antibodies including Smo (ab72130), E-cadherin (ab1416), N-cadherin (ab18203), MMP9 (ab76003), PCNA (ab92552) and Ki-67 (ab16667) were obtained from Abcam (UK).

### Cell culture

A2780 cells were obtained from ATCC (USA), whereas SKOV3 cells were purchased respectively from the Chinese Academy of Sciences Cell Bank (China). Ovcar8, HEK293T, and NIH3T3 cells were kindly provided by Professor Shiwen Luo. SKOV3, HEK293T, and NIH3T3 cells were cultured by DMEM, the A2780 and Ovcar8 cells were cultivated in RPMI 1640, and all these media were supplemented with 10% FBS (Excell, South America), and 1% penicillin/streptomycin (Solarbio, China). Cells were cultivated in accordance with the manufacturer’s guidelines.

### Plasmids and SMO^D473H^ mutagenesis

The Flag-Smo, Flag-Gli1, Myc-Gli2, 8×Gli-binding site luciferase reporter (pGL4.7-8×GBS-luciferase) plasmid, and pTK-Renilla luciferase plasmid were a kind gift from Professor Shiwen Luo. Flag-Smo^D473H^ point mutation assay followed the manufacturer’s protocol (SMK-101, Toyobo, Japan). Primers were as follows: F:5ʹ-CACTTCTTCAACCAGGCTGAGTGG - 3ʹ; R: 5ʹ- GTAGAAGTGGCAGCTGAAGGTAATGAG - 3ʹ.

### Cell proliferation and cytotoxicity assays

The EOC cells were inoculated onto 96-well plates at a density of 5×10^3^ cells, cultured for an overnight period, and then subjected to a variety of TPL (0–44.8 nM) concentrations. After incubation for 24, 48, or 72h, cell viability and cytotoxicity assays were conducted by Enhanced Cell Counting Kit-8 (Redmond, USA). Cell proliferation was assessed by measuring the optical density (OD) value at 450 nm. The nonlinear regression method was used in GraphPad Prism software 8.0 to calculate the 50% inhibitory concentration (IC_50_ values).

### EdU incorporation assay

For the purpose of assessing the proliferation of cancer cells, EdU staining was employed. The EOC cells (SKOV3:5×10^3^, A2780:7×10^3^, or Ovcar8:5×10^3^) were inoculated onto 96-well plates and cultured for an overnight period. TPL was applied to EOC cells at various dosages for 48 h, Subsequently, the EdU incorporation assay was analyzed following the manufacturer’s instructions (RiboBio Co., China), and visualized under a fluorescence microscope (Olympus, Japan), Data were quantified using ImageJ (National Institutes of Health, USA).

### Transwell migration and invasion assays

Cells (SKOV3: 8×10^3^, A2780: 1×10^4^ or Ovcar8: 8×10^3^) were added to the chamber (Corning, USA) with 200 μl serum-free medium and different doses of TPL with or without SAG. When they introduced, 600μl of medium supplemented with 10% FBS in the lower chamber. Subsequently, the cells were cultivated for 36 h at 37° C. The upper chamber was gently removed, the cells were fixed with 4% paraformaldehyde (POM) for 30 min, and stained with 0.1% crystal violet for 30 min, the non-migratory cells on the surface of the upper membrane were wiped with a cotton swab. Note that for the invasion experiment, the cells were seeded onto upper chambers coated with 50 ml (1:8) Matrigel (Sigma-Aldrich, C3867-1VL, USA). The images were captured with the light microscope in three random fields.

### Luciferase reporter assay

The HEK293T cells were planted and grown until they reached a density of 60-70%. Lipofectamine 3000 (Invitrogen, USA) was used to cotransfect the cells with the 8xGliBS-Luc (0.67 μg), pTK-Renilla (0.025 μg), and Gli (Gli1 or Gli2, 0.33 μg) plasmids. Cells were further cultivated for 36 h with Hh inhibitors (GANT61) or various concentrations of TPL after being cultured for 24 h. The subsequent process differs slightly different from HEK293T transfection, in that the NIH3T3 cells were cotransfected with the 8xGliBS-Luc and pTK-Renilla, stimulated with SAG with or without TPL or GANT61 for 36 h, and harvested for the luciferase assay according to the manufacturer’s protocol (Promega, USA), the firefly luciferase values were standardized to Renilla values.

### BODIPY-cyclopamine competition assay

To figure out whether Smo was the target of TPL, the BODIPY-cyclopamine competition assay was performed by microscopy and flow cytometry analyses. In brief, Lipofectamine 3000 was used to transfect the HEK293T cells with a Smo (1 μg) plasmid once they had attained 80% confluency. The cells were exposed to 5 nM BODIPY-cyclopamine (BioVision, USA) for 10 h supplemented with or without various concentrations of the compound after being cultured for 24 h. For the microscopy analysis, the cells will continue to be fixed in the dark with 4% paraformaldehyde (POM), incubated with 0.1% Triton X-100 for 10 min, and stained with DAPI solution for 10 min. Finally, the cells were observed and photographed with a fluorescence microscope. For the flow cytometry analysis, trypsin was used to harvest the cells, which were then fixed in the dark with 4% POM for 10 minutes before being incubated with 0.1% Triton X-100 for 10 minutes. Following that, the cells were resuspended in PBS for testing by flow cytometry (Agilent NovoCyte, USA). Data were analyzed by using the NovoExpress software 1.5.6 (Agilent, China).

### Cellular thermal shift assay (CETSA)

The CETSA experiment could be utilized to effectively understand the drug’s targets of action [59–61]. The principle of the CETSA experiment is that a target protein that binds to the drug molecule usually becomes stable and the amount of undegraded protein increases at the same temperature. Cells were incubated with DMSO or TPL (50 nM) for 2 h, harvested with trypsin, and resuspended in PBS containing phosphatase inhibitors. The cell was then aliquoted into PCR tubes and heated at the set temperatures (40, 44, 48, 52, 56, and 60° C) for 3 min, after cooling for 3 min. Subsequently, the protein was repeatedly frozen in liquid nitrogen and analyzed by Western blot.

### Immunofluorescence assay (IF)

The IF assay was constructed to assess the expression of Gli1 and Gli2 in the nucleus and cytoplasm after TPL (4nM) treatment. Cells were implanted in a 24-well plate covered with a round coverslip. After being adherent cells, using TPL or DMSO processing for 24 h, the cells were fixed in 4% POM, permeated with 0.1% Triton X-100, blocked with 10% BSA for 30 min, and incubated with anti-Gli (Gli1 or Gli2, 1:200 dilution) antibodies at 4° C for 12 h. Afterward, the cells were cultured with a secondary antibody (594 anti-Rabbit IgG, 1:200 dilution) along with DAPI at room temperature (RT) for 20min. The spherical coverslip was then gently removed and fixed. The images were captured with a laser scanning confocal microscope (Imager Vario LSM, Germany) with a ×63 objective in three random fields.

### Western blot assay

Cell’s total protein was lysed in RIPA containing 1% protease inhibitor, The protein concentration was measured quantitatively by BCA Protein assay kit (Thermo Scientific). Subsequently, the protein was denatured for 10 min with 100° C and loaded to conduct 8 or 10% SDS-PAGE electrophoresis for 2–3 h and transferred to an NC membrane for 1-3 h. The membrane was incubated with primary antibodies (anti-Gli1, anti-Ptch1, anti-Smo, anti-Bcl2, anti-E-cadherin, anti-N-cadherin and anti-MMP9, 1:1000 dilution) for 12 h at 4° C, anti-GAPDH (1:2500 dilution) antibody was used to perform normalization. After that, the membranes were incubated for 1 h at room temperature with a secondary antibody (anti-rabbit/mouse IgG, 1:1000 dilution). The immunoblot films were captured with an Epson V700 scanner.

### RT-qPCR

Trizol (Takara, China) was used to harvest the cell’s total RNA and the cDNA was generated from 1 μg total RNA using the of PrimeScript RT reagents Kit (TaKaRa, Japan). The RT-qPCR was performed by the M5 UltraSYBR One-Step RT-qPCR Kit (Mei5 Bioservices Co. Ltd., China). The primers for RT-PCR amplification were listed in Supplementary Table 1. The mRNA expression levels relative quantification was measured using the 2-ΔΔCT method.

### Immunoprecipitation and ubiquitination assay

The Myc-Ub (1 μg), Gli (GFP-Gli1 or GFP-Gli2, 2 μg), or GFP control vector plasmids were cotransfected into HEK293T cells. After being cultured for 24 h, the transfected cells were cultivated for 36 h with TPL. To prevent the degradation of ubiquitinated GFP-Gli1 or GFP-Gli2 from proteasomal degradation, MG132 was added for 4 h before harvesting protein. Part of the protein lysate was collected for the InPut group experiment, and the rest was incubated with MYC antibody overnight. The next day, ProteinA/G was blocked with 10% BSA for 30 min, and then 25 ul ProteinA/G beads were added to the protein lysate containing MYC antibody and incubated at 4° C for 3 h to eliminate the non-specifically binding proteins. The protein was denatured and loaded to conduct 8% SDS-PAGE electrophoresis for 6-8 h and transferred to an NC membrane for 3 h. The membrane was incubated with anti-GFP antibodies (1:1000 dilution) and anti-GAPDH antibodies (1:2500 dilution) for 12 h at 4° C, after that, the membranes were incubated for 1 h at room temperature with a secondary antibody (anti-rabbit/mouse IgG, 1:1000 dilution). Lastly, the immunoblot films were captured with an Epson V700 scanner.

### Animal modeling and treatment

We acquired BALB/c nude mice (4-6 weeks old, 14-16g) from the Nanjing Model Animal Research Center. After adaptive feeding for 7 days, the nude mice were subcutaneously inoculated with A2780 cells (200 mL, 8 × 10^6^ cells). The xenograft murine mice were randomly allocated into three groups (n=6) at random and intraperitoneally injected with DMSO, TPL (0.2 mg/kg/d), and TPL (0.4 mg/kg/d) once the tumor volume attained 50–100 mm3. Tumor volume was monitored with a caliper. The tumor volume (V) was measured by length × (width2) × 0.52. After 15 days, the mice were anesthetized (4% isoflurane) and sacrificed. The xenografted tumors were removed, weighed, and divided into two parts, one stored at -80° C and the other fixed in formalin for follow-up immunohistochemical (IHC) experiments. The major organs were collected and fixed in formalin. Animal-related experiments conformed to the Experimental Animal Science Center of Nanchang University and the Second Affiliated Hospital of Nanchang University Animal Care Principles (Approval Number: 0064257).

### Immunohistochemistry (IHC)

Xenografted tumors and major organs were fixed with formalin for 24 h and cut into 3.5 – 4 μm paraffin tissue blocks. Major organs were stained with H&E to evaluate drug toxicity. Briefly, the tumor sections were treated with citrate buffer (pH 6.0) for 2.5 min for antigen recovery, incubated with 3% H2O2 for 10 min, and then incubated with the antibodies (Ki-67, 1:500; PCNA, 1:50; Gli1, 1:250; Gli2, 1:200; Cleaved Caspase 3, 1:500 dilution) overnight at 4° C. Afterward, they were incubated with a secondary antibody for 1 h at 37° C, and then processed using a DAB kit (Cwbio, China) for 5–10 min, and finally counterstained with hematoxylin for 30s. The resulting immunolabeled sections were captured in images by using an FSX100 microscope (Olympus, Japan).

### Statistical analysis

The Student’s t-test or analysis of variance (ANOVA) was utilized for the statistical analysis and performed by GraphPad Prism 8.0 (GraphPad Software, USA). All outcomes were derived from three independent repeated experiments and shown as mean ± standard deviation (SD). P < 0.05 was considered statistically significant.

### Data availability statement

The original data are included in the article/Supplemental Material; further questions and all data are available from the corresponding author.

## Supplementary Material

Supplementary Figures

Supplementary Table 1
