# Supplementary material for: Triptolide inhibits epithelial ovarian tumor growth by blocking the hedgehog/Gli pathway
Source: Aging (Albany NY). 2023 Oct 17;15(20):11131–51. doi: 10.18632/aging.205110 (PMC10637820; doi:10.18632/aging.205110)
Supplement: Supplementary Figures [file aging-15-205110-s001.pdf]

SUPPLEMENTARY FIGURES

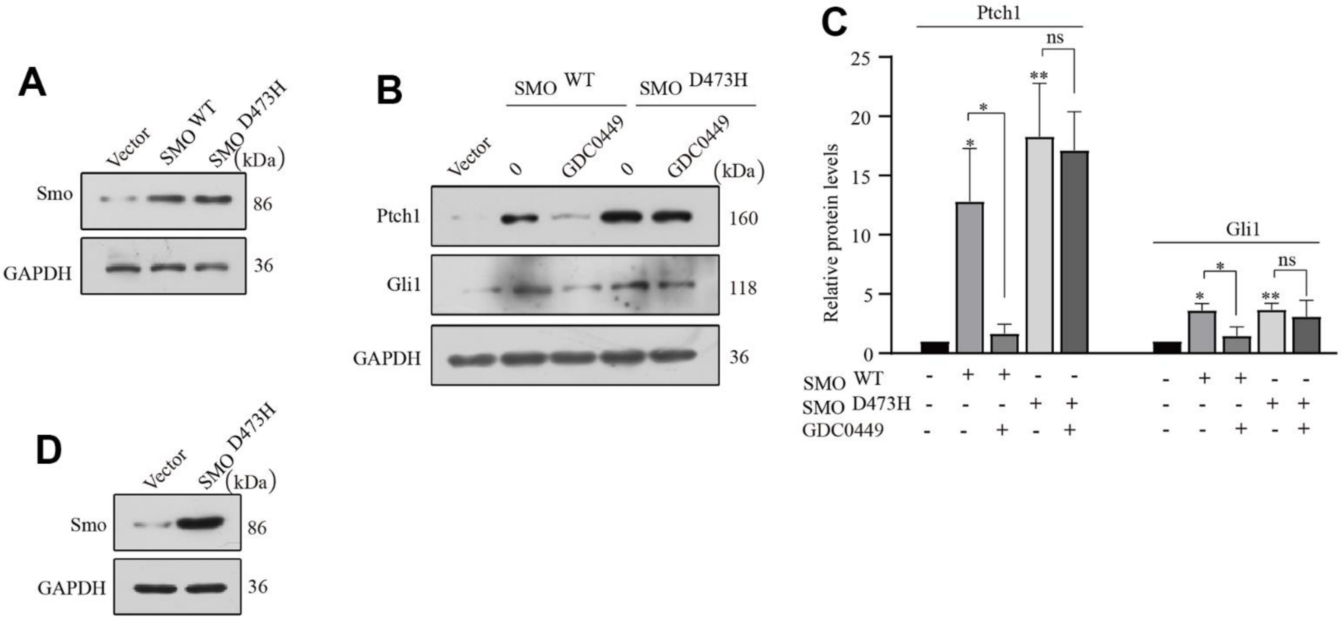

Supplementary Figure 1. SMOD^{473H} mutant plasmid verified by Western blot. (**A**) SMOD473H and SMOWT transfection efficiency being verified. (**B**, **C**) The mutant plasmid SMO473H was verified by GDC0449 (1 μM), and the SMOWT was used as the positive control. (**D**) Results of the manuscript's Figure 4D transfection efficiency being verified.

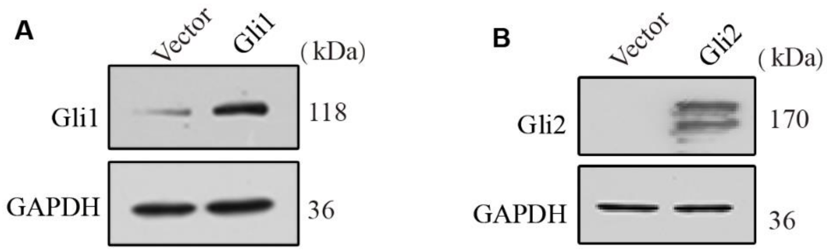

Supplementary Figure 2. The transfection efficiency was verified by Western blot. (**A**) Results of the manuscript's Figure 5C transfection efficiency being verified. (**B**) Results of the manuscript's Figure 5E transfection efficiency being verified.
