# Supplementary material for: Triptolide inhibits epithelial ovarian tumor growth by blocking the hedgehog/Gli pathway
Source: Aging (Albany NY). 2023 Oct 17;15(20):11131–51. doi: 10.18632/aging.205110 (PMC10637820; doi:10.18632/aging.205110)
Supplement: Supplementary Table 1 [file aging-15-205110-s002.pdf]

SUPPLEMENTARY TABLE

Supplementary Table 1. The information of primers.

Gene symbol	Senses (5′ -3′)	Senses (5′ -3′)
GAPDH	CACCAGGGCTGCTTTTAACTCTG	GATTTTGGAGGGATCTCGCTCCTG
Gli1	AGCGTGAGCCTGAATCTGTG	CAGCATGTACTGGGCTTTGAA
Gli2	CAGAATCGCACCCACTCCAACG	CGTGGACCGTTTTCACATGCTTCC
Ptch1	ACTCCCAAGCAAATGTACGAG	TTGAGTGGAGTTCTGTGCG
Bcl2	GCCCTGTGGATGACTGAGTACCTGAAC	CAGAGACAGCCAGGAGAAATCAAACAGAG
